# Safety and Efficacy of Indocyanine Green in Colorectal Cancer Surgery: A Systematic Review and Meta-Analysis of 11,047 Patients

**DOI:** 10.3390/cancers14041036

**Published:** 2022-02-18

**Authors:** Kamil Safiejko, Radoslaw Tarkowski, Tomasz Piotr Kozlowski, Maciej Koselak, Marcin Jachimiuk, Aleksander Tarasik, Michal Pruc, Jacek Smereka, Lukasz Szarpak

**Affiliations:** 1 Colorectal Cancer Unit, Maria Sklodowska-Curie Bialystok Oncology Center, 15-027 Bialystok, Poland; ksafiejko@onkologia.bialystok.pl (K.S.); tomaszpiotrkozlowski@gmail.com (T.P.K.); jumedica.onkologia@gmail.com (M.J.); olek.tarasik@gmail.com (A.T.); 2 Department of Surgical Oncology, Regional Specialist Hospital, 55-220 Legnica, Poland; rt@rakpiersi.net; 3 Institute of Outcomes Research, Maria Sklodowska-Curie Medical Academy, 03-411 Warsaw, Poland; Maciej.koselak@uczelniamedyczna.com.pl; 4 Oncological Surgery Subdivision, Masovian Oncology Hospital, 05-135 Wieliszew, Poland; 5 Research Unit, Polish Society of Disaster Medicine, 05-806 Warsaw, Poland; m.pruc@ptmk.org (M.P.); jacek.smereka@umed.wroc.pl (J.S.); 6 Laboratory for Experimental Medicine and Innovative Technologies, Department of Emergency Medical Service, Wroclaw Medical University, 51-616 Wroclaw, Poland

**Keywords:** indocyanine green (ICG), fluorescence, anastomotic leak, colorectal anastomoses, systematic review, meta-analysis

## Abstract

**Simple Summary:**

Indocyanine green (ICG) is a simple, inexpensive compound used in abdominal surgery, particularly advantageous in colorectal and rectal surgery, allowing intraoperative real-time assessment of the blood supply to the stumps of the large intestine after resection, and to the intestine after anastomosis in order to reduce the risk of anastomotic leak. We performed a systematic review and meta-analysis to evaluate the efficacy and safety of ICG in colorectal cancer surgery in a group of 11,047 patients. The anastomotic leak rate in the ICG and non-ICG groups varied and amounted to 3.7% vs. 7.6% (*p* < 0.001) in all trials, 8.1% vs. 12.1% (*p* = 0.04) in randomized controlled trials (RCTs), and 3.1% vs. 7.3% (*p* < 0.001) in non-RCTs, respectively. Our meta-analysis shows that ICG perfusion assessment, with its safety, simplicity, and short time of adjustment, is a tool worth considering in decreasing the rate of complications after colorectal surgery.

**Abstract:**

Despite the technological advances and improved surgical skills, the incidence of anastomotic leakage following colorectal cancer surgery still ranges from 4% to 19%. Therefore, we performed a systematic review and meta-analysis to evaluate the efficacy and safety of indocyanine green (ICG) use in colorectal cancer surgery. An online search of the Embase, MEDLINE, and Cochrane Central Register of Controlled Trials (CENTRAL) databases (from inception to 10 November 2021) was performed, in addition to manual screening. Thirty-two studies involving 11,047 patients were considered eligible for the meta-analysis. The anastomotic leak rate in the ICG and non-ICG groups varied and amounted to 3.7% vs. 7.6%, respectively (RR = 0.46; 95% CI: 0.39–0.56; *p* < 0.001). The rate in randomized controlled trials (RCTs) was 8.1% in the ICG group compared with 12.1% in the non-ICG group (RR = 0.67; 95% CI: 0.46–0.98; *p* = 0.04). In non-RCTs, it equaled 3.1% vs. 7.3%, respectively (RR = 0.43; 95% CI: 0.35–0.52; *p* < 0.001). Although the publications encompassed in our meta-analysis present different patients, with different factors influencing the results, a pooled analysis revealed a lower incidence of anastomotic leak in cases with ICG use. There are several other convincing advantages: safety, simplicity, and short time of the method adjustment. The presented meta-analysis indicates ICG perfusion assessment as a tool worth considering to decrease the rate of complications following colorectal surgery—valuable in the context of other, well-known risk factors.

## 1. Introduction

Indocyanine green (ICG) was approved for clinical use in 1959. It is an inexpensive, readily available, and simple-to-use compound characterized by low toxicity [1]. In the recent decade, owing to the rapid development of medical devices in surgery, including laparoscopic and robotic systems, the compound has found a variety of applications in abdominal organ surgery. The ICG technique has become particularly popular among surgeons performing colorectal surgery. It allows intraoperative real-time assessment of the blood supply to the stumps of the large intestine after resection and to the intestine after anastomosis [2]. Good blood supply to the anastomosis is crucial for proper healing and preventing intestinal anastomotic leak [3].

Anastomotic leak is one of the most serious colorectal and rectal surgery complications, significantly increasing postoperative morbidity and mortality. It also worsens the final oncological outcome and decreases the patients’ quality of life [4]. Currently available data show that the percentage of anastomotic leak in colorectal and rectal surgery ranges from 1% to 19%. Undoubtedly, the incidence of anastomotic leak is highly variable and depends on the anatomical location of the anastomosis, ranging 1–8% in ileocecal anastomoses and 5–19% in colorectal anastomoses [5,6].

It has been confirmed that the incidence of anastomotic leak in colorectal and rectal surgery depends on many factors. These are surgeon-dependent factors, such as the technique of anastomosis, blood supply to the anastomosis, lack of tension of the anastomosed bowel, timing of surgery, perioperative blood loss, restrictive perioperative fluid therapy, nutritional support, as well as surgeon-independent factors, such as age, male gender, comorbidities, malnutrition, obesity, stimulants, immunosuppression, preoperative chemotherapy and radiotherapy, advanced stage of cancer, and inflammatory bowel disease [5,7].

The most important factor influencing the proper healing of intestinal anastomosis is good blood supply. Other factors remain less significant if this condition is not fulfilled [8,9]. Therefore, it seems that intraoperative imaging of intestinal anastomotic blood supply by using ICG may become a predictive test of normal intestinal blood supply, which will consequently reduce the risk of anastomotic leak [10].

For this reason, we performed a systematic review and meta-analysis to evaluate the efficacy and safety of ICG use in colorectal cancer surgery.

## 2. Materials and Methods

The present systematic review and meta-analysis was performed in accordance with the Preferred Reporting Items for Systematic Reviews and Meta-Analyses (PRISMA) statement [11] (Table S3). Owing to the study design (meta-analysis), neither institutional review board approval nor patient informed consent were required.

### 2.1. Study Strategy

We conducted a systematic literature search of the Embase, MEDLINE, and Cochrane Central Register of Controlled Trials (CENTRAL) databases from inception to 10 November 2021. All references were saved in an EndNote (EndNote, Inc., Philadelphia, PA, USA) library used to identify duplicates. Terms related to ICG in colorectal cancer were applied: “Indocyanine Green” OR “indocyanine green-sulfo-OSu” OR “ICG” OR “Fluorescein Angiography” AND “Robotic Surgical Procedures” OR “Minimally Invasive Surgical Procedures” OR “laparosc*” OR “Rectum” OR “Rectal” OR “Colo-rect*” OR “surge*” OR “surgi*” OR “surgeo*” OR “Colorectal Surgery” OR “Ileostomy” OR “Colostomy” OR “Colectomy”. Additionally, we performed a manual search and review of the references listed in the retrieved articles.

### 2.2. Inclusion and Exclusion Criteria

The studies included in this meta-analysis met the following PICOS criteria: (1) Participants: adult patients requiring colorectal cancer surgery; (2) Intervention: surgery with ICG use; (3) Comparison: surgery without ICG use; (4) Outcomes: detailed information concerning anastomotic leak rate, occurrence of other adverse events; (5) Study design: randomized controlled trials (RCTs), retrospective trials. The exclusion criteria were as follows: (1) studies involving pediatric patients; (2) letters to the editor; (3) editorials; (4) conference abstracts; (5) guidelines.

### 2.3. Data Extraction

Two reviewers (M.P. and K.S.) independently extracted data from the identified eligible studies using a specifically designed data extraction form in Microsoft Excel^TM^ (Microsoft Corp., Redmond, WA, USA). Another author cross-checked these data before analysis (L.S.). The following data were extracted from each study: intraoperative parameters (i.e., operative time, intraoperative blood loss, transfusion requirement), postoperative parameters (i.e., anastomotic leak occurrence, adverse events). When there were suspected discrepancies in the data, we contacted the relevant authors directly.

### 2.4. Outcomes

The primary analysis focused on assessing anastomotic leak in patients undergoing colorectal cancer surgery with and without ICG application. Secondary outcomes included intraoperative parameters (i.e., operative time, intraoperative blood loss, transfusion requirement) and postoperative parameters (i.e., the occurrence of other adverse events, length of hospital stay, reoperation readmissions).

### 2.5. Risk of Bias Assessment

The risk of bias was determined independently by two reviewers (K.S. and M.K.); any disagreements were resolved by a third reviewer (L.S.). We applied revised tools to assess the risk of bias: RoB 2 for randomized trials [12] and ROBINS-I for non-randomized trials [13]. The overall RoB 2 judgment at the domain and study level was attributed in accordance with the criteria specified in the Risk-of-Bias VISualization (robvis) tool [14].

### 2.6. Statistical Analysis

The meta-analysis was entirely conducted with the Review Manager software, version 5.4 (Nordic Cochrane Center, Cochrane Collaboration, London, UK). The significance level for all statistical tests was *p* < 0.05 (two-tailed). Descriptive statistics are presented as numbers of cases (*n*) and percentages (%) for dichotomous and categorical variables or as means and standard deviations for continuous variables. When a continuous outcome was reported in a study as median, range, and interquartile range, we estimated means and standard deviations by using a formula described by Hozo et al. [15]. Heterogeneity was determined with the I^2^ statistic, in which the results range from 0% to 100%. Heterogeneity was interpreted as not observed with I^2^ = 0%, low with I^2^ = 25%, medium with I^2^ = 50%, and high with I^2^ = 75% [16,17]. A fixed model effect was applied when I^2^ < 50%, and a random model effect was used in other cases. For dichotomous data, we employed odds ratios (ORs) or risk ratios (RRs) as the effect measure with 95% confidence intervals (CIs); for continuous data, we used mean differences (MDs) with 95% CI. Subgroup analyses were performed with respect to study design (RCTs, non-RCTs.).

## 3. Results

### 3.1. Literature Search and Study Characteristics

**Figure 1:**
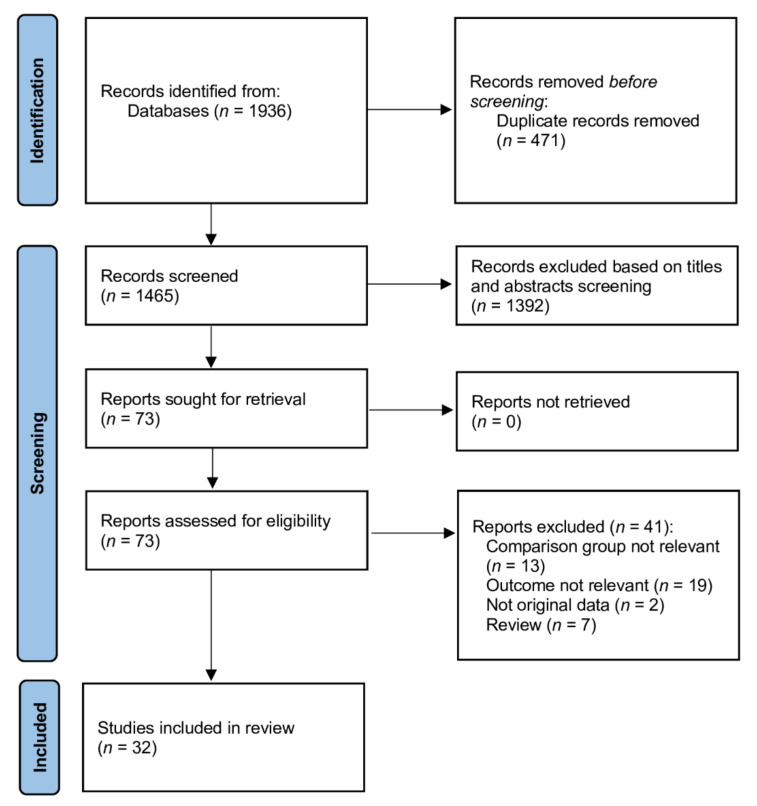
Database search and selection of studies in accordance with the PRISMA guidelines.

The detailed process of searching eligible studies is shown in Figure 1. A total of 1936 references were finally confirmed by the electronic search, and 73 potentially relevant studies were selected after screening the titles and abstracts. Among the 73 potentially relevant studies, a total of 32 met the inclusion criteria [18,19,20,21,22,23,24,25,26,27,28,29,30,31,32,33,34,35,36,37,38,39,40,41,42,43,44,45,46,47,48,49]. Table 1 and Table S1 summarize the study characteristics. The 32 included studies, involving 11,047 patients, were published between 2010 and 2021. The assessment of their risk of bias is provided in Figures S1–S4.

### 3.2. Intraoperative Evaluation

Seventeen studies reported operative duration, which equaled 214.9 ± 67.5 min in the ICG group and 228.9 ± 66.1 min in the non-ICG group (MD = −0.77; 95% CI: −12.42 to 10.87; I^2^ = 97%; *p* = 0.90).

Intraoperative blood loss was reported in eight studies and amounted to 128.7 ± 268.9 mL in the ICG group compared with 96.4 ± 135.8 mL in the non-ICG group (MD = −4.54; 95% CI: −17.43 to 8.35; I^2^ = 98%; *p* = 0.49).

Intraoperative transfusion was required in 1.4% of both ICG and non-ICG group patients (RR = 1.00; 95% CI: 0.37–2.72; I^2^ = 9%; *p* = 1.00). Additional sub-analyses by study design (RCTs and non-RCTs) are presented in Table S2.

### 3.3. Anastomotic Leak

**Figure 2:**
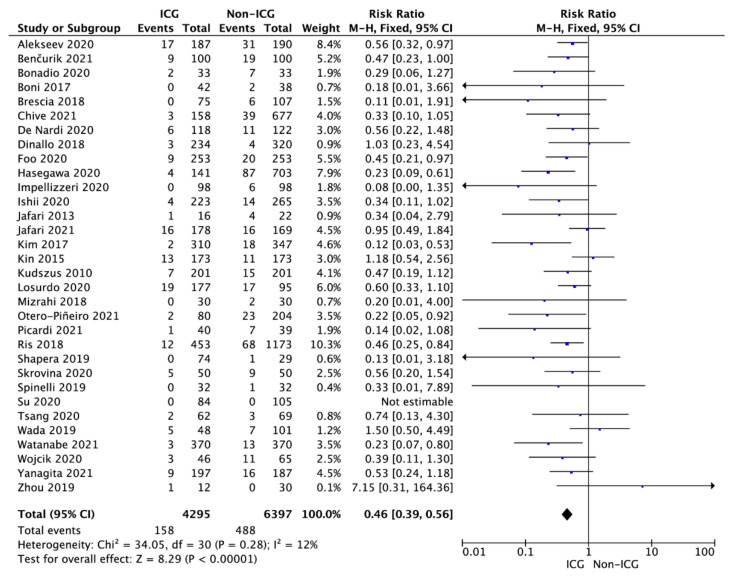
Forest plot of anastomotic leak occurrence in the ICG vs. non-ICG groups. The square centers represent the weighted risk ratios for individual trials, and the corresponding horizontal lines stand for the 95% CI. The diamonds represent pooled results. Legend: CI = confidence interval; ICG = indocyanine green.

The pooled analysis of the 32 studies showed that the overall anastomotic leak rate in the ICG and non-ICG groups varied and amounted to 3.7% vs. 7.6%, respectively (RR = 0.46; 95% CI: 0.39–0.56; *p* < 0.001; Figure 2).

**Figure 3:**
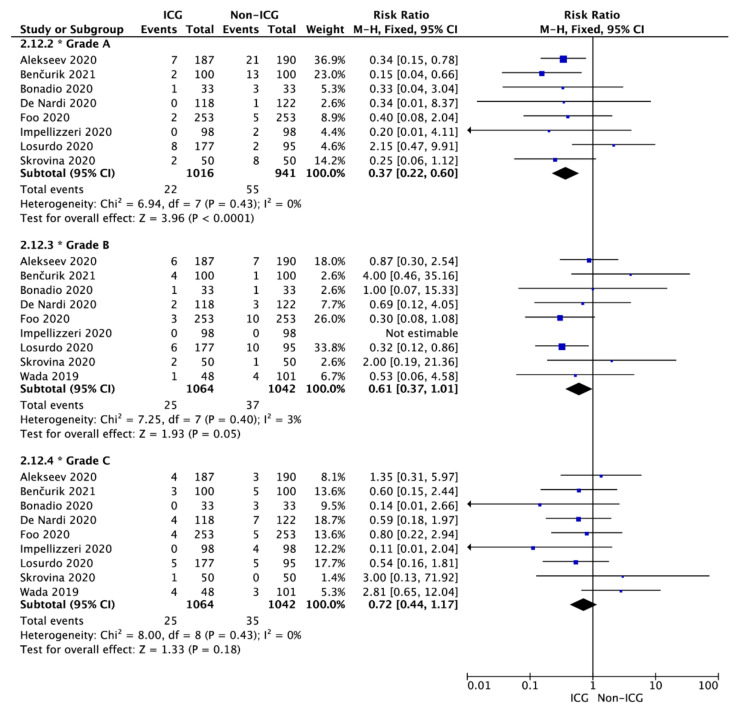
Forest plot of anastomotic leak occurrence in the ICG vs. non-ICG groups with sub-analysis with respect to the anastomotic leak grade. The square centers represent the weighted risk ratios for individual trials, and the corresponding horizontal lines stand for the 95% CI. The diamonds represent pooled results. Legend: CI = confidence interval; ICG = indocyanine green.

The same relationship was observed for the results as divided by grades of the anastomotic leak. The anastomotic leak rate was 2.2% vs. 5.8%, respectively, for grade A (RR = 0.37; 95% CI: 0.22–0.60; *p* < 0.001); 2.3% vs. 3.6% for grade B (RR = 0.61; 95% CI: 0.37–1.01; *p* = 0.05); and 2.2% vs. 3.4% for grade C (RR = 0.72; 95% CI: 0.44–1.17; *p* = 0.18; Figure 3).

The sub-analysis performed with respect to the type of study design showed an anastomotic leak rate of 8.1% in the ICG group compared with 12.1% in the non-ICG group (RR = 0.67; 95% CI: 0.46–0.98; *p* = 0.04) for RCTs [18,24,31] and a rate of 3.1% vs. 7.3%, respectively (RR = 0.43; 95% CI: 0.35–0.52; *p* < 0.001) for non-RCTs.

### 3.4. Postoperative Period Evaluation

A detailed list of the most frequently observed adverse events in the analyzed studies is presented in Table 2. Twelve studies reported the number of patients with postoperative adverse events. The pooled analysis revealed that in the ICG and non-ICG groups, postoperative adverse events incidence equaled 19.3% and 27.7%, respectively (RR = 0.80; 95% CI: 0.70–0.92; *p* = 0.002). However, the analyses of individual adverse events indicated no statistically significant differences between the ICG and non-ICG groups (*p* > 0.05).

Adverse events classified as Clavien–Dindo grade I–II varied in the ICG and non-ICG groups and amounted to 20.3% vs. 22.5% (RR = 0.91; 95% CI: 0.79–1.04; I^2^ = 50%; *p* = 0.15). In the case of grade III–IV, the corresponding values equaled 6.9% and 8.6% (RR = 0.88; 95% CI: 0.63–1.25; I^2^ = 0%; *p* = 0.49).

Thirteen studies reported the length of hospital stay, which was 8.7 ± 5.2 days for the ICG group compared with 8.5 ± 5.1 days for the non-ICG group (MD = −0.39; 95% CI: −0.84 to 0.05; I^2^ = 96%; *p* = 0.08).

The readmission rate in a 30-day follow-up was 4.6% vs. 7.2%, respectively, in the ICG vs. the non-ICG group (RR = 0.91; 95% CI: 0.48–1.70; I^2^ = 14%; *p* = 0.76) [23,37,43]. However, the readmission rate in a 60-day follow-up equaled 15.5% vs. 10.6% (RR = 0.85; 95% CI: 0.16–4.42; I^2^ = 61%; *p* = 0.85) [28,33,43].

The reoperation rate was reported in nine studies. It amounted to 3.4% in the ICG group compared with 5.3% in the non-ICG group (RR = 0.73; 95% CI: 0.47–1.12; I^2^ = 0%; *p* = 0.15).

## 4. Discussion

Disruption of anastomosis after colorectal surgery is still one of the most severe complications. Despite the progress in surgical oncology, its incidence reaches even 11–19% of patients [5,6,50,51,52]. According to Choi et al. [53], almost a third of patients with anastomotic insufficiency die for this reason. Several patient-dependent risk factors for anastomotic insufficiency following colorectal surgery include obesity, preoperative pelvis irradiation, male sex, malnutrition, low anastomosis, and tobacco use. The technical aspects crucial for proper anastomotic healing are the tension of the rectal stump and proximal part of the colon and appropriate blood circulation within the anastomosis. Blood support assessment helps estimate the risk and impacts on the level of proximal bowel transection [42]. Traditionally, such assessment is highly subjective and based on visible, active bleeding from the cut tissue and lack of discoloration of the bowel observed by the surgeon. A more sophisticated and effective method involves fluorescence scintigraphy, described aptly by Kudszus et al. [34] as “bringing light” into the (dark) picture of the anastomotic leakage. The PILLAR II study revealed insufficient blood circulation as assessed by ICG in 6.5% of patients in whom sufficient circulation was indicated in macroscopic evaluation [54]. The observation of impaired blood supply leads to modifications of the surgery through cutting the altered tissue at the level of proper microcirculation. This enables anastomosis healing and decreases the risk of anastomotic leak. Although ICG use demands an additional procedure, and poor microcirculation assessment results in additional bowel transection, our analysis did not imply any increase in the operation time, with the mean of 214.9 ± 67.5 min in the ICG group and 228.9 ± 66.1 min in the non-ICG group.

Our analysis demonstrated lower rates of anastomotic leak in both RCTs and non-RCTs.

A study by Otero-Piñeiro et al. [37], encompassing 284 patients with rectal cancer operated on with the transanal total mesorectal excision method with a mean anastomotic height of 4.85 vs. 5.08 cm and a diverting stoma constructed in 72.1% vs. 72.5% of patients, showed a modification of the surgical intervention after ICG angiography (ICGA) in 28.7% of cases. The authors noted 2.5% of anastomotic leak cases in the ICGA group vs. 11.3% in the control one (*p* = 0.02). ICGA was described as “an easy-to-use, accessible and reproducible technology that allows real-time evaluation of tissue perfusion”.

A meta-analysis by Blanco-Colino and Espin-Basany [10], involving 554 patients, showed a significant reduction of anastomotic insufficiency rate after the use of ICG fluoroscopy compared with interventions without its application (1.1% vs. 6.1%, respectively) and an 81% anastomotic insufficiency reduction owing to analyzed circulation assessment (OR = 0.19; 95% CI: 0.05–0.75; *p* = 0.02). In a multicenter study by Ris et al. [39], fluorescein angiography performed during surgery led to a change in the operation plan in 5.8% of cases. The PILLAR II study demonstrated no anastomotic leak incidence in the cases with alteration of the surgical plan after fluorescence angiography (7.9%, 11 cases) [54]. Other studies also indicated a lower rate of anastomotic insufficiency in cases of ICGA use for blood supply assessment. The publication by Skrovina et al. [41] showed an anastomotic leak incidence decrease by 8% (10% vs. 18% in the angioscintigraphy and the non-angioscintigraphy group), while fluoroscintigraphy extended the operation time by 6 min. ICG use changed the operation scenario in 12% of the patients when the resection line was moved from 2 to 5 cm proximally. In a single-surgeon retrospective study, Mizrahi et al. [36] reported even 0% incidence of anastomotic insufficiency in the group of 30 patients assessed with ICG. The examination changed the intervention in four (13.3%) individuals, while there were two anastomotic insufficiency cases in the control group (*n* = 30). It is worth mentioning that the average anastomosis level was low—2.8 cm from the anal verge; all of the patients were treated with a loop ileostomy. Similar results were obtained by Boni et al. [21]: there was a reduction of anastomotic insufficiency rate to 0% in the ICG group vs. 5% in the control group.

A lower rate of reoperations following intraoperative use of fluoroscintigraphy was reported by Kudszus et al. [34]: 3.5% vs. 7.5% in the control group. It is especially worth mentioning that the proportion was even more striking in the most vulnerable group of patients, i.e., those aged over 70 years (4.3% vs. 11.9% [*p* = 0.04]), in whom adverse events are associated with higher mortality. The risk of reoperation was reduced by 64% in this particular subgroup. Additionally, hospital stay was shortened in the fluorescence angiography group.

Fluorescence radiography and near-infrared angiography can help estimate blood microcirculation. However, a change of the transection line level owing to blood supply assessed as insufficient in fluorescein angiography did not prevent anastomotic leak in ther out of eighteen cases (16.7%) [48]. This could support the thesis by Kin et al. [33], implying that success is an effect of a combination of more than one factor concerning microcirculation.

Three randomized clinical trials assessed the effectiveness of ICG fluorescein angiography as the best available evidence source. Two of these support the hypothesis of the positive role of ICG fluorescein angiography in decreasing rates of anastomotic insufficiency, while one of them, despite the authors’ previous findings in a former phase II study, failed to demonstrate any difference between the outcomes in the examined groups. Our sub-analysis encompassing randomized clinical trials [18,24,31] showed an anastomotic leak rate of 8.1% in the ICG group and 12.1% in the non-ICG group (RR = 0.67; 95% CI: 0.46–0.98; *p* = 0.04).

The single-center FLAG trial performed by Alekseev et al. [18] encompassed 377 cases randomized to ICG fluorescein angiography or visual assessment of blood supply. Fluorescein angiography revealed impaired perfusion in 36 (19%) cases. Although anastomotic leak occurred in both groups, its rate was noticeably higher in the group without angiography: 31 (16.3%) vs. 17 (9.1%) in the ICG fluorescein angiography arm. The examination decreased anastomotic insufficiency rate in the group with low colorectal anastomoses (4–8 cm from the anal verge); the result was 14.4% vs. 25.7% in the non-ICG fluorescein angiography group (*p* = 0.04). There was no such effect among patients with higher levels of anastomosis (9–15 cm). ICG demonstrated impaired blood circulation in the bowel in 36 (19%) cases.

Another randomized trial, performed by De Nardi et al. [24], showed the favorable role of fluorescence angiography; it was, however, not statistically significant. Among 240 patients, angiography led to extended resection of the bowel in 11% of cases (*n* = 13), while anastomotic insufficiency occurred in six (5%) patients in the study group and eleven (9%) in the control one.

The PILLAR III study, performed by Jafari et al. [54], did not confirm the promising results of the PILLAR II study, showing the benefit of ICG application in decreasing the rate of anastomotic insufficiency after extensive bowel surgery. This RCT encompassed 347 patients in 25 centers and was concluded early owing to decreasing accrual rates. Its primary endpoint was the anastomotic leak; the secondary endpoints involved the effectiveness of perfusion assessment with ICG fluoroscopy and pelvic abscesses requiring surgical intervention. Both groups of patients had similar demographic characteristics, as well as tumor- and patient-dependent factors. Although perfusion was successfully assessed in 94.5% of cases, anastomotic insufficiency occurred in 9% of patients after ICG compared with 9.6% without the examined method usage. Surgical intervention was required in 6.9% of the perfusion group compared with 8.6% of the standard group. Postoperative abscess occurred in 5.7% of the intervention group and 4.2% of the control one. The rates of other complications were similar in both groups. The authors indicate the heterogeneity of groups assessed in the studies mentioned above compared with their material, i.e., the higher risk of anastomotic leak in their group due to preoperative radiotherapy in 65% of the patients compared with 10–20% in the other trials, a lower level of anastomosis, and a higher proportion of males. They explain the results with the multifactorial character of anastomotic insufficiency, the different surgeon experience, and the fact that the study was underpowered.

On the other hand, the distal rectal stump and the lack of its perfusion assessment appear to be the most critical factors. Since fluoroscintigraphy examines blood perfusion in the proximal part of the bowel, it does not assess blood circulation in the rectal stump. According to Vignali et al. [55], there is a positive correlation between anastomotic leak and altered blood flow in the rectal stump. On the contrary, Alexeev et al. [18] noted a “bright fluorescent reflection” in all cases. In a study by Kin et al. [33], ICG use did not change the rate of anastomotic leak, although it led to surgical management modification in some of the patients.

There are some weaknesses of the presented meta-analysis. The heterogeneity of the published material could impact on the results, showing the superiority of ICG angiography [45]. Heterogeneous operative methods (right and left hemicolectomy) were reported by Kudszus et al. [34] or Kin et al. [33], and small sample sizes characterized the studies by Jafari et al. [54] or Mizrahi et al. [36]. Moreover, some publications presented single-surgeon experience, and diverting stoma was formed in all patients described by Boni et al. [21]. Our review encompassed randomized clinical studies as the leading and most powerful evidence source, as well as retrospective, much smaller studies. The latter constituted the majority as prospective data are sparse.

## 5. Conclusions

The publications encompassed in our meta-analysis show different patients, with different factors influencing the results. The pooled analysis revealed a lower incidence of anastomotic leak in cases with ICG use. There are several other convincing advantages: safety, simplicity, and short time of the method adjustment. Although the results in the analyzed studies vary, the presented meta-analysis demonstrates ICG perfusion assessment as a tool worth considering to decrease the rate of complications following colorectal surgery—valuable in the context of other, well-known risk factors.

## Figures and Tables

**Table 1:** Characteristics of the included trials.

Study	Country	Study Design	Operation Method	ICG Group				Non-ICG Group			
				No.	Age (Years)	Sex, Male	AL Rate	No.	Age (Years)	Sex, Male	AL Rate
Alekseev et al., 2020 [18]	Russia	RCT	LAR, AR, LC	187	63 (21–86)	92 (49.2%)	17/187	190	63 (66–85)	92 (48.4%)	31 (16.3%)
Benčurik et al., 2021 [19]	Czech Republic	Retrospective	LAR with TME	100	62.6 ± 9.7	66 (66.0%)	9 (9.0%)	100	64.4 ± 9.2	64 (64.0%)	19 (19.0%)
Bonadio et al., 2020 [20]	Italy	Retrospective	RAR	33	71.85 ± 11.1	21 (63.6%)	2 (6.06%)	33	63.03 ± 11.3	15 (45.5%)	7 (21.21%)
Boni et al., 2017 [21]	Italy	Retrospective	LAR with TME	42	69 ± 8	28 (66.7%)	0 (0.0%)	38	67 ± 7	22 (57.9%)	2 (5.3%)
Brescia et al., 2018 [22]	Italy	Retrospective	CL, LAR, ACR	75	67.1 ± 6	43 (57.3%)	0 (0.0%)	107	65.7 ± 7	63 (58.9%)	6 (5.6%)
Chivé et al., 2021 [23]	France	Retrospective	CL, PR	158	64 ± 15	95 (60.1%)	3 (1.9%)	677	62 ± 16	374 (55.2%)	39 (5.8%)
De Nardi et al., 2020 [24]	Italy	RCT	LAR, CL	118	66.1	60 (50.8%)	6 (5.1%)	122	65.1	66 (54.1%)	11 (9.0%)
Dinallo et al., 2019 [25]	USA	Retrospective	LAR	234	61.5 (34.6–88.4)	108 (46.2%)	3 (1.3%)	320	62.5 (35.3–89.7)	138 (43.1%)	4 (1.3%)
Foo et al., 2020 [26]	China	Retrospective	TME	253	66.6 ± 10.6	(65.6%)	3.6%	253	67.2 ± 11.0	64.4%	7.9%
Hasegawa et al., 2020 [27]	Japan	Retrospective	LAR, ISR	141	63 (51–69)	99 (70.2%)	4 (2.8%)	703	62 (55–68)	450 (0%)	87 (12.4%)
Impellizzeri et al., 2020 [28]	Italy	Retrospective	LAR, LSH, SR	98	66 (59–74)	53 (54.1%)	0 (0.0%)	98	71 (58–79)	57 (58.2%)	6 (6.1%)
Ishii et al., 2020 [29]	Japan	Retrospective	Mixed	233	67 (30–90)	126 (43.1%)	4 (1.8%)	265	69 (27–93)	136 (51.3%)	14 (5.3%)
Jafari et al., 2013 [30]	USA	Retrospective	LAR, ISR	16	58	12 (75.0%)	1 (6.3%)	22	63	16 (73%)	4 (18.2%)
Jafari et al., 2021 [31]	USA	RCT	LAR	178	57.2 ± 11.4	109 (61.2%)	16 (9.0%)	169	57.0 ± 11.4	99 (58.6%)	16 (9.6%)
Kim et al., 2017 [32]	Korea	Case cohort	LAR	310	58 ± 11	182 (58.9%)	2 (0.6%)	347	57 ± 11	216 (62.2%)	18 (5.2%)
Kin et al., 2015 [33]	USA	Retrospective	CL, PR	173	58.2 ± 13.2	54 (31.2%)	13 (7.5%)	173	58.1 ± 13.2	54 (31.2%)	11 (6.4%)
Kudszus et al., 2010 [34]	Germany	Retrospective	HC	201	67.8 ± 25.2	85 (42.2%)	7 (3.5%)	201	69.0 ± 21.9	85 (42.2%)	15 (7.5%)
Losurdo et al., 2020 [35]	France	Retrospective	CR	177	69.9 ± 11.2	109 (61.4%)	19 (10.8%)	95	67.9 ± 10.0	37 (38.6%)	17 (17.8%)
Mizrahi et al., 2018 [36]	USA	Retrospective	LAR	30	58 ± 12	16 (53.3%)	0 (0.0%)	30	58 ± 13	18 (60.0%)	2 (6.7%)
Otero-Piñeiro et al., 2021 [37]	Spain	Retrospective analysis of prospectively collected data	TaTME	80	68.0 ± 11.4	51 (63.7%)	2 (2.5%)	204	66.6 ± 12.3	123 (60.3%)	23 (11.3%)
Picardi et al., 2021 [38]	Italy	Retrospective	Mixed	40	62.6 ± 10.5	17 (42.5%)	1 (2.5%)	39	67.74 ± 13.4	19 (48.7%)	7 (17.9%)
Ris et al., 2018 [39]	Multicenter	Prospective open-label clinical study	Mixed	504	64 (18–88)	279 (55.4%)	0 (0.0%)	1173	NS	NS	68 (5.8%)
Shapera et al., 2019 [40]	USA	Prospectively maintained database	LAR, HC, SI	74	58	42 (56.8%)	0 (0.0%)	29	60	16 (55.2%)	1 (3.4%)
Skrovina et al., 2020 [41]	Czech Republic	Retrospective	TME	50	62.4 ± 9.0	34 (68.0%)	5 (10.0%)	50	65.0 ± 9.4	29 (58.0%)	9 (18.0%)
Spinelli et al., 2019 [42]	Italy	Retrospective	IPAA	32	39.41 ± 14.09	21 (65.6%)	0 (0.0%)	32	45.75 ± 15.9	17 (53.1%)	1 (3.12%)
Su et al., 2020 [43]	China	Retrospective	CL	84	59.1 ± 11.1	48 (57.1%)	0 (0.0%)	105	60.2 ± 9.8	55 (52.4%)	0 (0.0%)
Tsang et al., 2020 [44]	China	Prospective	LAR, HC, AR	62	69.82 ± 9.89	39 (62.9%)	2 (3.2%)	69	67.71 ± 11.65	47 (68.1%)	3 (4.3%)
Wada et al., 2019 [45]	Japan	Retrospective	LAR	48	66	31 (64.6%)	5 (10.4%)	101	67	70 (69.3%)	7 (6.9%)
Watanabe et al., 2021 [46]	Japan	Retrospective	SSSA	532	74 (68–80)	273 (51.3%)	2/260 (0.8%)	502	73 (66–79)	268 (44.4%)	7/274 (2.6%)
Wojcik et al., 2020 [47]	France	Prospective	CL, AR	46	65.7 ± 11.1	30 (65.2%)	3 (6.5%)	65	68.6 ± 12	40 (61.5%)	11 (16.9%)
Yanagita et al., 2021 [48]	Japan	Retrospective analysis of prospectively collected data	Mixed	197	70 (34–93)	116 (58.9%)	9 (4.6%)	187	69 (38–94)	115 (61.5%)	16 (8.6%)
Zhou et al., 2019 [49]	China	Retrospective	TME	12	60.3 ± 9.6	5 (41.7%)	1 (8.3%)	30	58.5 ± 9.5	19 (63.3%)	0 (0.0%)

Legend: ACR = atypical colonic resection; AR = anterior resection; CL = colectomy; CR = colorectal resection; HC = hemicolectomy; ICG = indocyanine green; IPAA = ileal pouch–anal anastomosis; ISR = intersphincteric resection; LAR = low anterior resection; LSH = laparoscopic supracervical hysterectomy; NS = not stated; PR = proctectomy; RCT = randomized controlled trial; SI = sigmoidectomy; SR = sigmoid resection; SSSA = stapled side-to-side anastomosis; TaTME = transanal total mesorectal excision; TME = total mesorectal excision.

**Table 2:** Pooled analysis of adverse events in the included trials.

Adverse Event Type	No. of Studies	Events/Participants		Events		Heterogeneity between Trials		*p* Value for Differences across Groups
		ICG	Non-ICG	RR	95% CI	*p* Value	I^2^ Statistic	
No. of patients with adverse events	12	218/1129 (19.3%)	376/1358 (27.7%)	0.80	0.70–0.92	0.11	36%	0.002
Wound infection	13	34/1401 (2.4%)	53/1615 (3.3%)	0.72	0.47–1.09	0.83	0%	0.12
Ileus	12	64/1381 (4.6%)	91/1624 (5.6%)	0.90	0.67–1.23	0.06	43%	0.51
Abdominal bleeding	4	6/401 (1.5%)	11/538 (2.0%)	1.02	0.38–2.79	1.00	0%	0.96
Abdominal abscess	4	7/266 (2.6%)	18/442 (4.1%)	0.83	0.36–1.92	0.60	0%	0.66
Bowel obstruction	2	4/182 (2.2%)	1/203 (0.5%)	3.32	0.50–21.85	0.39	0%	0.21
Urinary retention	10	23/829 (2.8%)	34/1112 (3.1%)	0.88	0.51–1.50	0.90	0%	0.63
Urinary tract infections	6	17/774 (2.2%)	125/301 (41.5%)	0.77	0.43–1.37	0.56	0%	0.37
Urinary injury	2	1/70 (1.4%)	1/69 (1.4%)	0.99	0.14–6.83	0.33	0%	0.99
Pulmonary complications	7	26/678 (3.8%)	34/760 (4.5%)	0.86	0.53–1.38	0.31	15%	0.53
Cardiovascular complications	2	2/128 (1.6%)	1/128 (0.8%)	1.00	0.18–5.62	0.37	0%	1.00

Legend: CI = confidence interval; ICG = indocyanine green; RR = risk ratio. Note: Not all outcomes were reported in every study. “No. of studies” refers to the studies included in the analysis for the particular outcome.

## Supplementary Materials

The following supporting information can be downloaded at: https://www.mdpi.com/article/10.3390/cancers14041036/s1. Table S1: Methodology characteristics of the included trials [18,19,20,21,22,23,24,25,26,27,28,29,30,31,32,33,34,35,36,37,38,39,40,41,42,43,44,45,46,47,48,49]; Table S2: Pooled analysis in subgroups of randomized and non-randomized trials; Table S3: PRISMA checklist. Figure S1: A summary table of the review authors’ judgements for each risk of bias item for each randomized study; Figure S2: A plot of the distribution of the review authors’ judgements for each risk of bias item across randomized studies; Figure S3: A summary table of the review authors’ judgements for each risk of bias item for each non-randomized study; Figure S4: A plot of the distribution of the review authors’ judgements for each risk of bias item across non-randomized studies.
