# Supplementary material for: Safety and Efficacy of Indocyanine Green in Colorectal Cancer Surgery: A Systematic Review and Meta-Analysis of 11,047 Patients
Source: Cancers (Basel). 2022 Feb 18;14(4):1036. doi: 10.3390/cancers14041036 (PMC8869881; doi:10.3390/cancers14041036)
Supplement: Supplementary file 1 [file cancers-14-01036-s001.zip › cancers-1528469-supplementary.pdf]

cancers

MDPI

Supplementary Material

Safety and Efficacy of Indocyanine Green in Colorectal Cancer Surgery: a Systematic Review and Meta-Analysis of 11,047 Patients

CONTENTS:

Table S1. Methodology characteristics of the included trials. 2
Table S2. Pooled analysis in subgroups of randomized and non-randomized trials. 10
Table S3. PRISMA checklist. 11
Figure S1. A summary table of the review authors' judgements for each risk of bias item for each randomized study. 14
Figure S2. A plot of the distribution of the review authors' judgements for each risk of bias item across randomized studies. 14
Figure S3. A summary table of the review authors' judgements for each risk of bias item for each non-randomized study. 15
Figure S4. A plot of the distribution of the review authors' judgements for each risk of bias item across non-randomized studies. 16

Table S1. Methodology characteristics of the included trials.

Study	Inclusion criteria	Exclusion criteria	ICG dose	ICG imaging system	Primary outcome(s)	Findings
Alekseev et al. 2020	Elective patients with either malignant or benign sigmoid or rectal neoplasms who were the candidates for resection with a stapled colorectal anastomosis located less than 15 cm from the anal verge with or without diverting ileostomy.	Known allergy to iodine and impaired kidney function.	0.2 mg/kg	Laparoscopic system (KARL STORZ GmbH &Co. KG, Tuttlingen, Germany) with a light source (D-LIGHT P SCB; KARL STORZ)	To assess whether ICG FA was associated with a reduction in the incidence of AL.	ICG FA is associated with a reduction in AL following low anterior resection.
Benčurik et al. 2020	Patients with rectal cancer, with or without neoadjuvant therapy, who were indicated for low anterior rectal resection with TME with primary double-stapled anastomosis, using mini-invasive techniques	Open or converted operations for rectal cancer, patients with synchronous and recurring colon cancer and patients with an allergy to iodine contrast solutions.	0.2 mg/kg	SPIES system (KARL STORZ GmbH & Co. KG, Tuttlingen, Germany) or Firefly™ robotic surgical system da Vinci Xi (Intuitive Surgical, Sunnyvale, CA, USA).	To assess the effectiveness of intraoperative fluorescence angiography (FA) in decreasing the incidence of AL after minimally invasive low anterior resection (LAR) with total mesorectal excision (TME) in rectal cancer patients and to determine predictors of anastomotic leak.	The use of ICG to visualize tissue perfusion in low rectal resections for cancer can lead to a reduction of AL.
Bonadio et al. 2020	All the patients subjected to elective laparoscopic RAR for extraperitoneal rectal cancer	All patients operated on in an emergency setting or with open technique.	0.2 mg/kg	Laparoscopic SPIES system (KARL STORZ GmbH&Co. KG, Tuttlingen, Germany) and a full high-definition camera system (IMAGE 1 SPIESTM, KARL	To evaluate the efficacy of this tool in patients subjected to elective laparoscopic RAR for extraperitoneal rectal cancer.	The use of ICGf seems to have great potential for reducing AL rates in rectal surgery. Our experience showed a downward trend in AL rates in the ICGf setting, thus

				STORZ). A xenon light source was employed (D-LIGHT P SCB, KARL STORZ),		confirming the possible benefits and safety of this new technology.
Boni et al. 2016	All patients undergoing LAR for cancer with total mesorectal excision (TME) followed by colorectal or coloanal anastomosis	Patients undergoing transanal TME, with iodine allergy, as well as pregnant and/or lactating women	5 cc of 0.2 mg/kg	The Karl Storz image fluorescence system (Karl Storz, Tuttlingen, Germany)	Postoperative complications classed according to the Dindo–Clavien classification.	In our experience, ICG FA was safe and effective in low rectal cancer resection, possibly leading to a reduction in the anastomotic leakage rate after TME.
Brescia et al. 2018	Patients underwent elective laparoscopic colorectal procedures	NS	0.25 mg/kg)	Laparoscopic SPIES system (KARL STORZ GmbH & Co.KG, Tuttlingen, Germany); The imaging was generated by the high-end full HD camera system (IMAGE 1 SPIES™ system, KARL STORZ GmbH & Co.KG, Tuttlingen, Germany) connected to a laparoscope with 30° field of direction and 10 mm diameter in all cases. A xenon light source (D-LIGHT P SCB, KARL STORZ)	anastomotic leakage rate, length of stay, readmission rate, and mortality between the two groups	The combination between ICG and ERAS protocol is feasible and safe, with a statistically significant reduction of the AL when combined in colorectal surgery.
Chive et al. 2021	Patients that underwent elective colorectal resection (right, left, transverse or total colectomy and proctectomies, with or without a diverting stoma) with anastomosis	NS	0.5 mg/kg	The camera (a Photodynamic Eye PC6100 C9830−10 from Hamamatsu, Hamamatsu, Japan, or a Novadaq from Stryker, Kalamazoo, MI)	incidence of AL	Intraoperative fluorescence imaging with ICG is associated with a lower incidence of AL
De Nardi et al. 2019	Adult patients who were referred for laparoscopic anterior rectal resection or left colectomy with colorectal anastomosis located between 2 and 15 cm from the anal verge, for either malignant or benign disease, provided that ligation of the inferior mesenteric artery at its origin or immediately	Known ICG or iodine allergy, chronic kidney disease stage 3, 4, or 5, pregnancy or lactation, and patients undergoing abdominoperineal rectal excision or limited sigmoid resection without ligation of the inferior mesenteric artery.	0.3 mg/kg two times	KARL STORZ GmbH & Co. KG, Tuttlingen, Germany	incidence of AL	Intraoperative ICG fluorescent angiography can effectively assess vascularization of the colic stump and anastomosis in patients undergoing colorectal resection. This method led to further proximal bowel resection in 13 cases, however, there was no statistically significant reduction of

	above the left colic artery was performed.					anastomotic leak rate in the ICG arm.
Dinallo et al. 2018	All colorectal resections at a single institution performed with intra-operative ICG-FA	NS	2mL of ICG	SPY Elite System (Stryker, USA), Pinpoint System (Stryker, USA)	incidence of AL	No significant difference was found in anastomotic leak rates between the two groups studied. Routine use of fluorescence angiography significantly altered intra-operative decision-making without discernible change in clinical outcome
Foo et al. 2020	Patients undergoing elective left-sided colorectal resections	Multi-visceral resections, pelvic exenterations and total proctocolectomy with ileal pouch- anal anastomosis	Bolus of 5 mg. For the Pinpoint and the Da Vinci Xi Firefly system, the dosage of ICG was 7.5 mg.	SPY Elite System (Stryker, USA), Pinpoint System (Stryker, USA) and the Da Vinci Xi (Firefly, Intuitive Surgical, Sunnyvale, CA, USA).	incidence of AL	The routine use of ICG FI was associated with a lower anastomotic leakage rate in anterior resections. The reduction in anastomotic leakage rate was mainly seen in TME.
Hasegawa et al. 2020	Patients who underwent elective laparoscopic low anterior resection (LAR) or intersphincteric resection (ISR) with lymphadenectomy for malignant rectal tumors using ICG-FA	History of left-sided colorectal surgery or simultaneous resection of other primary cancers	5.0 mg ICG	IMAGE1 S™ system (Karl Storz SE & Co. KG, Tuttlingen, Germany), 1588 Advanced Imaging Modalities (AIM) Platformand SPY Fluorescence technology (Stryker, Kalamazoo, MI, USA), or HyperEye Medical System Handy (Mizuho Medical Co. Ltd., Tokyo, Japan	incidence of AL	Intraoperative ICG-FA is a promising method to reduce anastomotic leakage after laparoscopic rectal surgery.
Impellizzeri et al. 2020	Patients who underwent colorectal surgery. Left-sided hemicolectomy, sigmoid resection, and anterior rectal resection were included.	Age younger than 18 years, right-sided hemicolectomy (RH), transverse colon resection, atypical resections, and colorectal surgical procedures performed in an emergency setting.	5 ml bolus of diluted ICG (25 mg of ICG)	IMAGE 1 SPIESTM, KARL STORZ	incidence of AL	The use of the NIRF/ICG was safe for colorectal surgery and decreases the risk of anastomotic leak.

Ishii et al. 2019	Patients with colorectal cancer who underwent laparoscopic colorectal resection with creation of an anastomosis	Previous history of colorectal resection, allergic hypersensitivity to ICG or iodine, multiorgan resection, preoperative intestinal obstruction, more than two anastomoses due to multiple sites of colorectal cancer, intra-abdominal infection, or severe comorbidities	5-mg	NS	Incidence of AL	ICG fluorescence angiography can potentially reduce the AL rate with laparoscopic rectal cancer surgery.
Jafari et al. 2013	Rectal cancer cases treated surgically via robot-assisted low and ultralow anterior resection, as well intersphincteric resection (ISR)	Patients with permanent colostomy	2 mg/kg	Olympus Corporation (Tokyo, Japan), Karl Storz GmbH (Tuttlingen, Germany), Stryker Corporation (Portage, MI, USA), and Novadaq Technologies (Ontario, Canada).	Incidence of AL	ICG fluorescence may play a role in anastomotic tissue perfusion assessment and affect the AL rate.
Jafari et al. 2021	18 years of age or older; undergoing open, or minimally invasive LAR for the treatment of a rectal or rectosigmoid Neoplasm; rectal or rectosigmoid neoplasm(s) may be treated with or without neoadjuvant therapy. Long-course neoadjuvant therapy must have been completed ≥6 weeks prior to LAR surgery (Day 0); planned low circular stapled or transanally hand sewn anastomosis ≤10 cm from the anal verge; colorectal or coloanal reconstruction with or without reservoir/pouch; women of child-bearing potential must not be pregnant or lactating, must have a negative pregnancy test at Day 0; signed an approved informed consent form for the study; willing to comply with the protocol.	Undergoing stapled anastomosis with the use of an experimental or non-FDA approved stapler. 2. Undergoing ileoanal reconstruction, total colectomy or proctocolectomy, abdominoperineal resection, Hartmann's procedure, Hartmann's reversal or multiple synchronous colon resections (e.g., LAR and concomitant right colectomy). 3. Has received and completed a course of pelvic radiotherapy ≥ 6 months prior to LAR surgery (Day 0). 4. Has previously undergone a left sided colon resection. 5. Has previously undergone a rectal resection. 6. Has recurrent rectal or rectosigmoid cancer. 7. Has a diagnosis of locally advanced rectal or rectosigmoid cancer undergoing extended en bloc operations. 8. Has a diagnosis of Stage IV rectal or rectosigmoid cancer with any multifocal metastases or single site metastasis with tumor size of > 2 cm a. Intraoperative incidental finding or preoperative suspicion of Stage IV cancer with isolated (single site) metastasis (≤ 2 cm) or limited metastases (≤3), with	3.0 ± 1.0 mL of a 2.5-mg/mL concentration (for proximal colon) 3.0 ± 1.0 mL of a 2.5-mg/mL solution (for transanal imaging)	PINPOINT and/or SPY Elite near infrared range fluorescence imaging (Stryker, Kalamazoo, MI)	Incidence of AL	Successful visualization of perfusion can be achieved with indocyanine green fluoroscopy. However, no difference in anastomotic leak rates was observed between patients who underwent perfusion assessment versus standard surgical technique. In experienced hands, the addition of routine indocyanine green fluoroscopy to standard practice adds no evident clinical benefit.

		largest lesion ≤ 2 cm in size, does not exclude the subject. 9. Has a diagnosis of inflammatory bowel disease (IBD). Subjects with rectal or rectosigmoid cancer neoplasms and IBD are excluded. 10. Has hepatic dysfunction defined as Model for End-Stage Liver Disease (MELD) Score >12. 11. Renal dysfunction defined as creatinine ≥ 2.0 mg/dL. 12. Has known allergy or history of adverse reaction to ICG, iodine or iodine dyes. 13. Has, in the Investigator's opinion, any medical condition that makes the subject a poor candidate for the investigational procedure, or interferes with the interpretation of study results. Is actively participating in another investigational clinical study which, in the Investigator's or Sponsor's opinion, would interfere in this study				
Kim et al. 2017	Patients with rectal cancer, who underwent curative robot-assisted sphincter-saving operations	NS	10 mg per procedure, 25 mg per vial	the da Vinci Si or Xi system (Firefly, Intuitive Surgical, Sunnyvale, CA	To quantitatively define the indocyanine green fluorescent imaging findings based on perfusion (mesocolic and colic) time and perfusion intensity (5 grades) to provide probable indications.	Quantitative analysis of indocyanine green fluorescent imaging may help prevent anastomotic complications during robot-assisted sphincter-saving operations, and may be of particular value in high-class ASA patients, older patients, and patients with a short descending mesocolon.
Kin et al. 2015	Patients ≥18 years of age who underwent elective colon or rectal resections with a primary anastomosis	Perineal proctectomies, ileocolic and ileorectal anastomoses, and total proctocolectomy with IPAAs	3 mL	SPY Imaging System (Novadaq Technologies Inc, Bonita Springs, FL)	anastomotic leak occurring within 60 days of the initial operation	Intraoperative fluorescence angiography to assess the perfusion of the colon conduit for anastomosis was not associated with colorectal anastomotic leak. Perfusion is but one of multiple factors contributing to anastomotic leaks.
Kudszus et al. 2010	All anastomosis or resection margins in colorectal cancer resections were	NS	0.2–0.5 mg/kg	System (IC-View®, Pulsion Medical Systems AG, Munich, Germany)	Incidence of AL	There was an overall reduction in the absolute revision rate of 4% in the LFA group and a significantly

	investigated intraoperatively using LFA					reduced rate of revision in the subgroup analysis of patients undergoing elective colorectal resections, in patients older than 70 years and in patients with hand-sewn anastomosis. This demonstrates that LFA is a method that may significantly reduce not only the rate of severe complications in colorectal surgery but also the hospital length of stay.
Losurdo et al. 2020	Patients undergoing rectal and left colon cancer surgery.	Patients operated for benign disease in which we use a different vascular approach with a low ligation of the inferior mesenteric artery; patients that were operated in urgency with a laparotomic approach and all patients that were operated with laparotomic approach for other reasons. patients in whom the operation was converted from laparoscopic to open surgery	0.2 mg/kg	SPIES system for a laparoscopic procedure (Karl Storz, Germany) and a full HD camera system (Karl Storz Image 1-Professional Image Enhancement System-SPIESTtm, Karl Storz,Germany). A xenon light source was employed (D-Light P system, Karl Storz Endoscope®),	incidence of AL	Hypoperfusion is a well-recognized cause of AL. The ICG assessment of colic vascularization is a simple, inexpensive, and side effects free method, which can sensibly reduce both overall AL and type B and type C fistulas when routinely used.
Mizrahi et al. 2018	All patients who underwent an elective laparoscopic LAR for a rectal neoplasm with a colorectal or coloanal anastomosis < 5 cm from the anal verge	A planned laparotomy, a redo coloanal anastomosis, an urgent or emergent operation, and/or anastomosis > 5 cm from the anal verge, or a planned abdominoperineal resection.	0.1–0.3 mg/kg	PINPOINT™ Endoscopic Fluorescence Imaging System (Novadaq, Toronto, Ontario, Canada)	AL occurring within 60 days of the initial operation	Fluorescence angiography changed the surgical plan in 13.3% of LAR's, potentially reducing the incidence of AL in these high-risk patients.
Otero-Piñeiro et al. 2020	Adult patients diagnosed with primary rectal cancer, undergoing elective curative TaTME with planned colorectal/coloanal anastomosis	Patients treated with abdominoperineal resection or Hartmann, pregnant or breastfeeding women, and those with non-adenocarcinoma tumors. Patients with known allergy to ICG or iodine and patients treated with iodine dyes or drugs known to interact with ICG (anticonvulsants, drugs containing bisulfite, methadone, nitrofurantoin).	2.5 mg/ml	PINPOINT™ Endoscopic Fluorescence Imaging System (Novadaq, Toronto, Ontario, Canada)	Incidence of AL during the first 30 days after index surgery.	ICG fluorescence angiography modified the proximal colonic transection in more than one-quarter of patients, leading to a significant decrease of AL rate.
Picardi et al. 2021	Patients underwent colorectal surgery	NS	0.3-0.4 mg/ml/kg	Laparoscopic system (KARL STORZ GmbH & Co. KG, Tuttlingen, Germany) with the	Incidence of AL	The study confirms the validity of the use of ICG fluorescence as a method for intraoperative

				image generated by a Full HD camera (IMAGE 1 SPIESTM, KARL STORZ) connected to a laparoscope at 30 degrees and 10 mm in diameter with a specific filter for NIR fluorescence. The light source (D-LIGHT P SCB, KARL STORZ)		assessment of bowel perfusion even in emergency conditions and in acute postoperative hemorrhage, detecting an incidence of 2.5% (1 case out of 40) of AL. It's evident that to validate our results, further randomized studies on a larger data set are required. It would also be beneficial to evaluate quantitatively the fluorescence between the mucous and serous layer, to confirm the reduction of AL rate, the better evaluation of bowel perfusion and, especially in emergency surgeries, the potential reduction of further operations
Ris et al. 2018	All patients aged over 18 years scheduled for elective colorectal surgery involving resection and anastomosis of any type	Failure to meet the inclusion criteria; pregnancy or lactation; previous adverse reaction or allergy to ICG or iodine; significant liver dysfunction; emergency surgery; refusal to participate after informed consent was sought; and lack of availability of the NIR-ICG laparoscope owing to recent use/need for sterilization.	2,5mg /ml (3 ml per injection)	PINPOINT® Endoscopic Fluorescence Imaging System (Stryker, Kalamazoo, Michigan, USA)	incidence of AL	Routine NIR-ICG assessment in patients undergoing elective colorectal surgery is feasible. NIR-ICG use may change intraoperative decisions, which may lead to a reduction in anastomotic leak rates.
Shapera et al. 2019	Patients underwent left-sided robotic-assisted colorectal anastomosis by a single colorectal surgeon for various indications.	NS	25mg	Vinci Xi robot (Intuitive Surgical),	Incidence of AL	IcGA is safe to use as demonstrated by the very low rate of complications in this case series. It is also safe to rely on to guide reresection and recreation of an anastomosis intraoperatively by demonstration of blood flow
Skrovina et al. 2020	Patients aged over 18 years of age, who underwent elective low rectal resection for cancer with total mesorectal excision, with primary double stapling end-to-end anastomosis and protective ileostomy	Patients with rectal cancer recurrence, those acutely operated or those with converted minimally invasive operations, as well as patients with a known allergy to iodine substances and pregnant or breastfeeding patients	0.2 mg/kg.	SPIES system (KARL STORZ GmbH & Co. KG, Tuttlingen, Germany) or the Firefly robotic surgical system da Vinci Xi (Intuitive Surgical, Sunnyvale, CA, USA).	The effectiveness of fluorescence angiography using ICG in decreasing the incidence of anastomotic leak of the colorectal anastomosis	Fluorescence angiography using indocyanine green is a safe and effective method with the potential of reducing anastomotic leak during minimally invasive low resection of the rectum for cancer.

	by laparoscopic or robotic technique					
Spinelli et al. 2019	Patients undergoing IPAA	NS	0.2–0.1 mg/kg	PINPOINT endoscopic fluorescence imaging system (Stryker, Kalamazoo, Michigan, USA), a laparoscopic Spies system (Karl Storz GmbH, Tuttlingen, Germany) and a full high definition camera system (Image 1 SPIESTM, Karl Storz), with xenon light source (D-Light P SCB, Karl Storz).	Incidence of AL	FA is applicable to IPAA surgery and may help to reduce perfusion-related anastomotic leaks.
Su et al. 2020	18–80 years of age, body mass index (BMI) between 18.5 and 30 kg/m2 and with a pathological diagnosis of colon adenocarcinoma by colonoscopy	Patients with history of past colonic surgery, multiple colorectal primary carcinomas, distant metastasis, and allergic hypersensitivity to ICG.	Minimum dose of 3 ml	opto-cam 2100 (Optomedic, Guangdong, China).	Incidence of AL	IGFI shows promise as a safe and feasible tool to assess bowel perfusion during a totally laparoscopic surgery for colon cancer and may reduce the operative time.
Tsang et al. 2020	All patients underwent elective colorectal operations	Age < 18 years, pregnancy, emergency surgery, surgery without anastomosis (such as abdominoperineal resection, Hartmann's operation etc.) and history of adverse resection or allergy to ICG.	10 mg	Da Vinci Xi robotic surgical system (Intuitive Surgical, Sunnyvale, CA, USA) or Olympus laparoscopic camera system OTV-S300 with IR light source CLV-S200-IR (Olympus, Tokyo, Japan).	Anastomotic leakage rate within 30 days from surgery	ICG fluorescent imaging is a feasible and safe tool to assess colonic vascularisation for patients undergoing colorectal surgery. However, it did not significantly lower the anastomotic leakage rate. ICG should not be routinely used in colorectal surgery before an available large scale randomised controlled trial to prove any clinical benefits.
Wada et al. 2018	Rectal cancer patients underwent elective laparoscopic LAR with DST anastomosis	Patients with allergic sensitivity to iodine. cases with a protective stoma	5 mg	NIR camera system (PDE-neo System; Hamamatsu Photonics K.K., Hamamatsu, Japan)	Incidence of AL	Intraoperative ICG angiography is useful for prediction of AL following laparoscopic LAR.
Watanabe et al. 2021	1) had histologically proven colon cancer; 2) had undergone D2 or D3 dissection according to the Japanese Classification of Colorectal	1) a history of treatment for other abdominal malignancy; 2) reconstruction other than antiperistaltic SSSA (eg, handsewn anastomosis and triangle anastomosis using a linear stapler); 3) multiple	0.25 mg/kg	Stryker Corporation (1588 AIM Platform; Kalamazoo, MI), Olympus Medical Systems Corporation (VISERA ELITE	The percentage of anastomotic leak within 30 days after surgery.	This large-scale case-matched study showed that assessing perfusion by near-infrared observation significantly reduced the anastomotic

	Carcinoma; and 3) had undergone antiperistaltic SSSA, also known as functional end-to-end anastomosis	primary cancers; and 4) emergent cases.		II; Tokyo, Japan), and Karl Storz (D-Light P; Tuttlingen, Germany)		leak and reoperation rates after stapled side-to-side anastomosis in colon cancer surgery and may be better suited to colo-colonic anastomosis.
Wojcik et al. 2020	All consecutive adult patients who underwent a left colectomy or anterior resection for cancer and who had a colorectal or coloanal anastomosis	Colorectal resections for cancer with concomitant cytoreductive surgery	0.1 mg/kg	NIR light images (FLUOBEAM ; Fluoptics, Grenoble, France) or on fusion images merging NIR and standard white light images (PINPOINT ; Stryker, Kalamazoo, Michigan, USA).	The percentage of anastomotic leak within 30 days after surgery.	In this prospective case-matched study, IOFA decreased the occurrence of clinically relevant AL due to necrosis of the descending colon or anastomosis. Upon blind review, perfusion assessment using IOFA was reproducible.
Yanagita et al. 2021	Patients with left-sided colon or rectal cancer who underwent ICG fluorescence imaging during elective surgery	Surgery without anastomosis, such as Hartmann's operation or abdominoperineal resection, and patients with a history of allergic reaction to ICG and/ or iodine.	0.1 mg/kg	Near-infrared excitation light (we used mainly Hyper Eye Medical Systems: Mizuho Medical Co., Ltd, Nagoya, Japan and/or IMAGE 1 SPIES™, KARL STORZ SE & Co. KG, Tuttlingen, Germany)	Incidence of AL	Even though this study has limitations of comparison of data prospectively collected and retrospectively analyzed, intraoperative ICG fluorescence imaging evaluation could significantly decrease the incidence of AL.
Zhou et al. 2019	(1) Patients with rectal adenocarcinoma confirmed by endoscopic biopsy; (2) Patients with suspected LPNM based on magnetic resonance imaging (MRI) evaluation; and (3) The tumour was located under the retroperitoneum (within 8 cm of the anal margin).	(1) recurrent patients and; (2) patients with distant metastasis.	0.1 mg/mL	Near-infrared camera system (Karl Storz Endoskope spies TM GmbH & Co. KG, Tuttlingen, Germany)	Incidence of AL	ICG-enhanced NIR fluorescence-guided imaging could be a feasible and convenient technique to guide LPND because it could bring specific advantages regarding the accuracy and completeness of surgery as well as safety.

Table S2. Pooled analysis in subgroups of randomized and non-randomized trials

PARAMETER	RCT				Non-RCT			
	No. of studies	Events RR / MD	Events 95%CI	P-value for differences across groups	No. of studies	Events RR / MD	Events 95%CI	P-value for differences across groups
Operative duration	2	9.87	1.80 to 17.95	0.02	15	1.14	-0.28 to 2.55	0.12
Intraoperative blood loss	1	6.70	-3.50 to 16.90	0.20	7	-7.23	-21.83 to 7.38	0.33
Intraoperative transfusion rate	0	-	-	-	3	1.68	0.40 to 7.06	1.00
Overall anastomoses leak	3	0.67	0.46 to 0.98	0.04	29	0.43	0.35 to 0.52	<0.001
Grade A	2	0.34	0.15 to 0.76	0.008	6	0.38	0.20 to 0.72	0.003
Grade B	2	0.82	0.33 to 2.04	0.66	7	0.54	0.30 to 0.98	0.04
Grade C	2	0.82	0.33 to 2.05	0.67	7	0.68	0.39 to 1.21	0.19
Claiven Dindo I-II	1	0.85	0.44 to 1.63	0.62	7	0.86	0.61 to 1.21	0.39
Hospital length of stay	2	3.22	-3.15 to 9.59	0.32	11	-0.81	-1.24 to -0.38	<0.001
36-d readmission rate	0	-	-	-	3	0.85	0.16 to 4.42	0.85
Reoperation rate	1	0.69	0.07 to 6.94	0.75	8	0.73	0.47 to 1.13	0.16
**ADVERSE EVENTS**								
No of patients with adverse events	1	0.93	0.55 to 1.59	0.80	11	0.79	0.69 to 0.91	0.001
Wound infection	3	0.50	0.19 to 1.30	0.16	10	0.79	0.50 to 1.25	0.31
Ileus	3	1.72	0.81 to 3.65	0.15	9	0.79	0.57 to 1.11	0.18
Abdominal bleeding	3	1.13	0.23 to 5.48	0.88	1	0.96	0.52 to 1.84	0.94
Abdominal abscess	0	-	-	-	4	0.83	0.36 to 1.92	0.66
Bowel obstruction	0	-	-	-	2	3.32	0.50 to 21.85	0.21
Urinary retention	3	0.64	0.22 to 1.83	0.41	7	0.98	0.52 to 1.84	0.94
Urinary tract infections	1	1.38	0.22 to 8.75	0.74	5	0.72	0.39 to 1.33	0.29
Urinary injury	0	-	-	-	2	0.99	0.14 to 6.83	0.99
Pulmonary complications	1	0.15	0.01 to 2.83	0.20	6	0.94	0.58 to 1.54	0.81
Cardiovascular complications	0	-	-	-	2	1.00	0.18 to 5.62	1.00

Table S3. PRISMA checklist

Section and Topic	Item #	Checklist item	Location where item is reported
**TITLE**			
Title	1	Identify the report as a systematic review.	1
**ABSTRACT**			
Abstract	2	See the PRISMA 2020 for Abstracts checklist.	1
**INTRODUCTION**			
Rationale	3	Describe the rationale for the review in the context of existing knowledge.	2
Objectives	4	Provide an explicit statement of the objective(s) or question(s) the review addresses.	2
**METHODS**			
Eligibility criteria	5	Specify the inclusion and exclusion criteria for the review and how studies were grouped for the syntheses.	2,3
Information	6	Specify all databases, registers, websites, organisations, reference lists and	2

Section and Topic	Item #	Checklist item	Location where item is reported
sources		other sources searched or consulted to identify studies. Specify the date when each source was last searched or consulted.	
Search strategy	7	Present the full search strategies for all databases, registers and websites, including any filters and limits used.	2,3
Selection process	8	Specify the methods used to decide whether a study met the inclusion criteria of the review, including how many reviewers screened each record and each report retrieved, whether they worked independently, and if applicable, details of automation tools used in the process.	3
Data collection process	9	Specify the methods used to collect data from reports, including how many reviewers collected data from each report, whether they worked independently, any processes for obtaining or confirming data from study investigators, and if applicable, details of automation tools used in the process.	2,3
Data items	10a	List and define all outcomes for which data were sought. Specify whether all results that were compatible with each outcome domain in each study were sought (e.g. for all measures, time points, analyses), and if not, the methods used to decide which results to collect.	3
	10b	List and define all other variables for which data were sought (e.g. participant and intervention characteristics, funding sources). Describe any assumptions made about any missing or unclear information.	3
Study risk of bias assessment	11	Specify the methods used to assess risk of bias in the included studies, including details of the tool(s) used, how many reviewers assessed each study and whether they worked independently, and if applicable, details of automation tools used in the process.	3
Effect measures	12	Specify for each outcome the effect measure(s) (e.g. risk ratio, mean difference) used in the synthesis or presentation of results.	3
Synthesis methods	13a	Describe the processes used to decide which studies were eligible for each synthesis (e.g. tabulating the study intervention characteristics and comparing against the planned groups for each synthesis (item #5)).	3
	13b	Describe any methods required to prepare the data for presentation or synthesis, such as handling of missing summary statistics, or data conversions.	3
	13c	Describe any methods used to tabulate or visually display results of individual studies and syntheses.	3
	13d	Describe any methods used to synthesize results and provide a rationale for the choice(s). If meta-analysis was performed, describe the model(s), method(s) to identify the presence and extent of statistical heterogeneity, and software package(s) used.	3

Section and Topic	Item #	Checklist item	Location where item is reported
	13e	Describe any methods used to explore possible causes of heterogeneity among study results (e.g. subgroup analysis, meta-regression).	3
	13f	Describe any sensitivity analyses conducted to assess robustness of the synthesized results.	3
Reporting bias assessment	14	Describe any methods used to assess risk of bias due to missing results in a synthesis (arising from reporting biases).	3
Certainty assessment	15	Describe any methods used to assess certainty (or confidence) in the body of evidence for an outcome.	3
**RESULTS**			
Study selection	16a	Describe the results of the search and selection process, from the number of records identified in the search to the number of studies included in the review, ideally using a flow diagram.	4
	16b	Cite studies that might appear to meet the inclusion criteria, but which were excluded, and explain why they were excluded.	4
Study characteristics	17	Cite each included study and present its characteristics.	4-6
Risk of bias in studies	18	Present assessments of risk of bias for each included study.	4
Results of individual studies	19	For all outcomes, present, for each study: (a) summary statistics for each group (where appropriate) and (b) an effect estimate and its precision (e.g. confidence/credible interval), ideally using structured tables or plots.	4-9
Results of syntheses	20a	For each synthesis, briefly summarise the characteristics and risk of bias among contributing studies.	6-9
	20b	Present results of all statistical syntheses conducted. If meta-analysis was done, present for each the summary estimate and its precision (e.g. confidence/credible interval) and measures of statistical heterogeneity. If comparing groups, describe the direction of the effect.	4-9
	20c	Present results of all investigations of possible causes of heterogeneity among study results.	6-9
	20d	Present results of all sensitivity analyses conducted to assess the robustness of the synthesized results.	6-9
Reporting biases	21	Present assessments of risk of bias due to missing results (arising from reporting biases) for each synthesis assessed.	6
Certainty of evidence	22	Present assessments of certainty (or confidence) in the body of evidence for each outcome assessed.	6

Section and Topic	Item #	Checklist item	Location where item is reported
**DISCUSSION**			
Discussion	23a	Provide a general interpretation of the results in the context of other evidence.	9
	23b	Discuss any limitations of the evidence included in the review.	12
	23c	Discuss any limitations of the review processes used.	12
	23d	Discuss implications of the results for practice, policy, and future research.	12
**OTHER INFORMATION**			
Registration and protocol	24a	Provide registration information for the review, including register name and registration number, or state that the review was not registered.	-
	24b	Indicate where the review protocol can be accessed, or state that a protocol was not prepared.	2
	24c	Describe and explain any amendments to information provided at registration or in the protocol.	2
Support	25	Describe sources of financial or non-financial support for the review, and the role of the funders or sponsors in the review.	12
Competing interests	26	Declare any competing interests of review authors.	12
Availability of data, code and other materials	27	Report which of the following are publicly available and where they can be found: template data collection forms; data extracted from included studies; data used for all analyses; analytic code; any other materials used in the review.	12

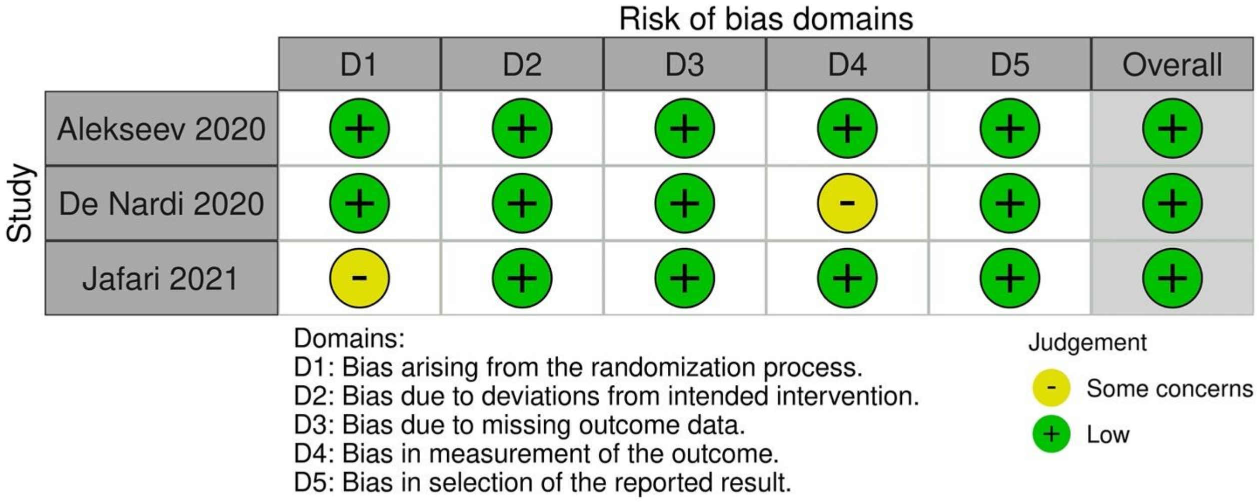

Figure S1. A summary table of the review authors' judgements for each risk of bias item for each randomized study.

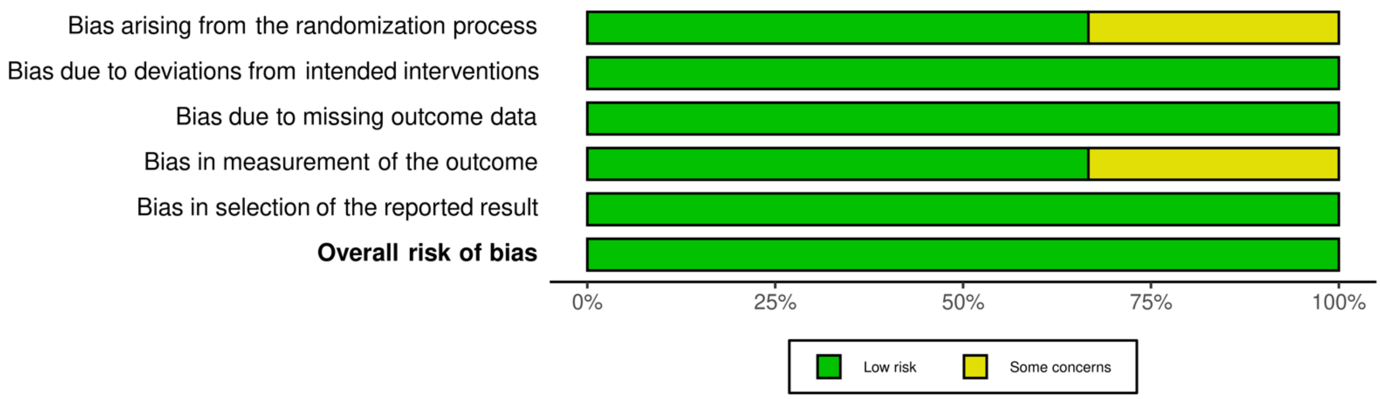

Figure S2. A plot of the distribution of the review authors' judgements for each risk of bias item across randomized studies.

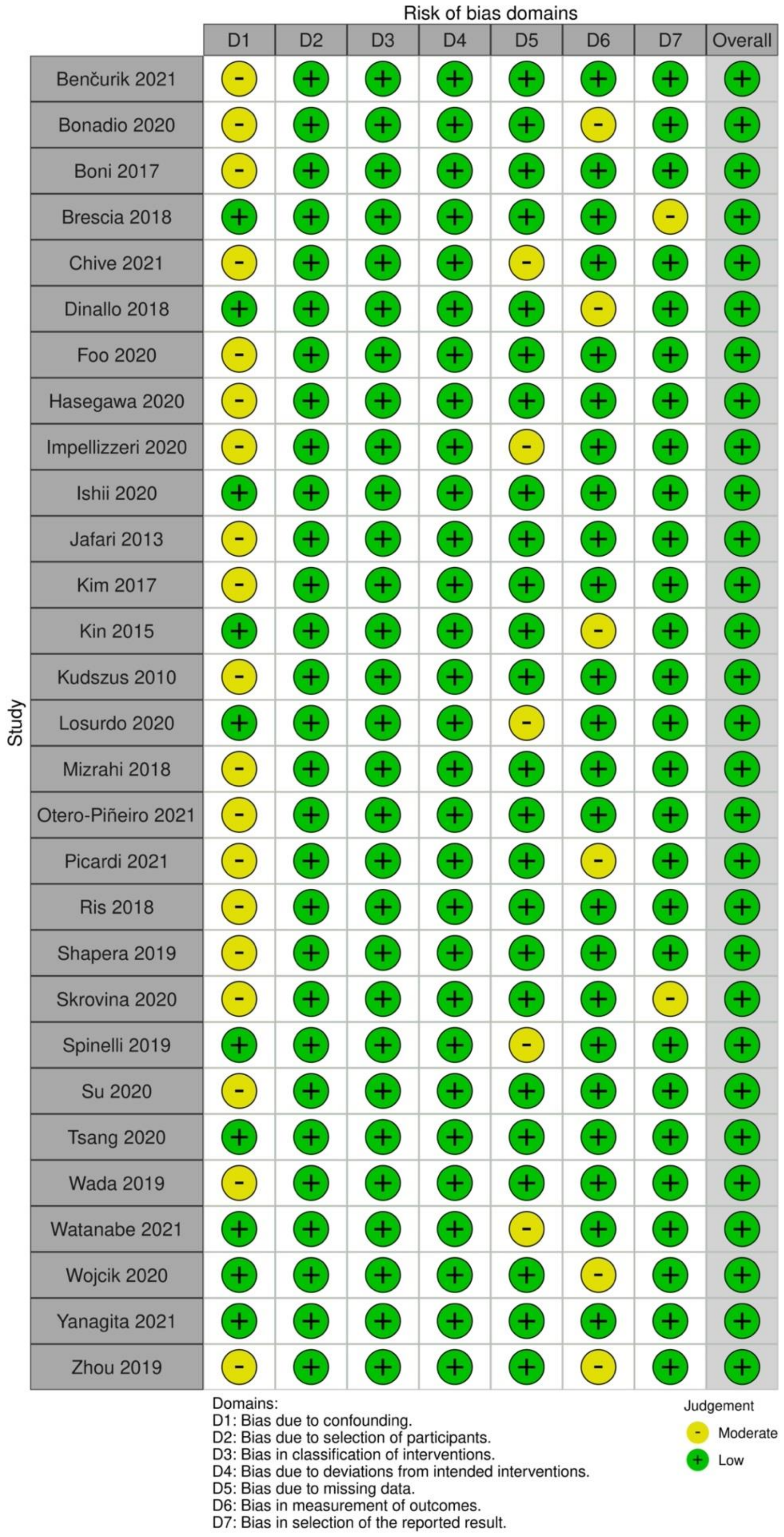

Figure S3. A summary table of the review authors' judgements for each risk of bias item for each non-randomized study.

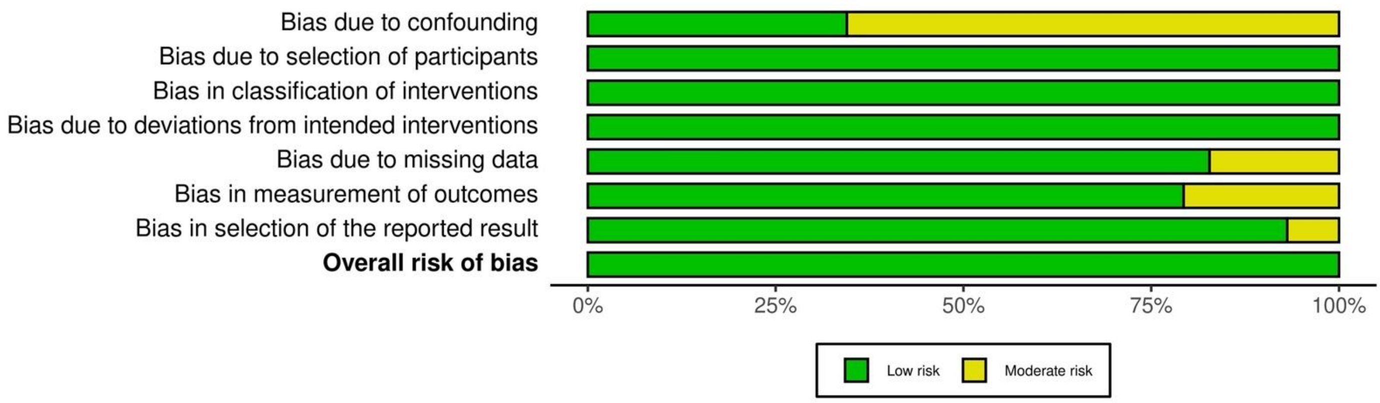

Figure S4. A plot of the distribution of the review authors' judgements for each risk of bias item across non-randomized studies.
